# Immune profiling identifies CD8^+^ T-cell subset signatures as prognostic markers for recurrence in papillary thyroid cancer

**DOI:** 10.3389/fimmu.2022.894919

**Published:** 2022-11-07

**Authors:** Zhen Chen, Meng-Li Guo, Ya-Yi Li, Kai Yan, Liang Li, Fei Shen, Haixia Guan, Qing-Zhi Liu, Bo Xu, Zhe-Xiong Lian

**Affiliations:** ^1^ Department of Thyroid Surgery, the Second Affiliated Hospital, School of Medicine, South China University of Technology, Guangzhou, China; ^2^ Department of Thyroid Surgery, Guangzhou First People’s Hospital, Guangzhou Medical University, Guangzhou, China; ^3^ Department of Thoracic Surgery, Guangdong Provincial People’s Hospital, Guangdong Academy of Medical Sciences, Guangzhou, Guangdong, China; ^4^ Guangdong Provincial People’s Hospital, Guangdong Academy of Medical Sciences, Guangzhou, Guangdong, China; ^5^ Department of Endocrinology, Guangdong Provincial People’s Hospital, Guangdong Academy of Medical Sciences, Guangzhou, Guangdong, China; ^6^ The Second School of Clinical Medicine, Southern Medical University, Guangzhou, Guangdong, China; ^7^ Chronic Disease Laboratory, School of Medicine, South China University of Technology, Guangzhou, Guangdong, China

**Keywords:** papillary thyroid cancer (PTC), recurrence, CD8^+^T cells, multiplex immunohistochemistry, PD-1, CD39, CD103

## Abstract

**Background:**

Thyroid tissue has a special immune microenvironment that is not well characterized. Whether immune cells have a prognostic value in the recurrence of papillary thyroid cancer (PTC) needs further investigation.

**Methods:**

Multinodular non-toxic goiter (MNG) was taken as normal tissue for the difficulty in obtaining completely normal thyroid tissue (normal thyroid function, no thyroiditis, and no nodules). We compared the composition of mononuclear cells (MNCs) in peripheral blood and thyroid tissues from MNG and PTC patients by high-dimensional flow cytometry profiling and verified the results by multiplex immunohistochemistry. The recurrence rates of PTC patients with different CD8^+^T cell subset signatures were compared using TCGA database.

**Results:**

We observed that the immune cell composition of MNG was different from that in peripheral blood. Thyroid tissue contains higher percentages of T cells and NK cells. Moreover, the percentages of memory T cells and Treg cells were higher in thyroid than in peripheral blood and increased in PTC tumors. We further focused on the antitumoral CD8^+^T cells and found that the expression patterns of PD-1, CD39, and CD103 on CD8^+^T cells were different between MNG and PTC. Importantly, we found higher percentages of PD-1^+^CD39^+^CD103^+^CD8^+^T and PD-1^+^CD39^+^CD103^-^CD8^+^T cells in PTC tumor tissues from recurrent patients than non-recurrent patients. By analyzing PTC data from TCGA database, we found that the expression patterns of these molecules were associated with different pathologic types and genders among PTC patients. Moreover, patients with PD-1^hi^CD39^lo^CD103^hi^CD8^hi^, PD-1^hi^CD39^hi^CD103^lo^CD8^hi^, and PD-1^lo^CD39^hi^CD103^hi^CD8^hi^ expression patterns have a higher 10-year recurrence-free survival.

**Conclusion:**

The immune microenvironment in MNG tissue is distinct from that in peripheral blood and paratumor tissue. More memory CD8^+^T cells were detected in PTC, and expression patterns of PD-1, CD39, and CD103 on CD8^+^T cells were significantly different in physiology and gender and associated with the recurrence rate of PTC. These observations indicate that CD8^+^T cell signatures may be useful prognostic markers for PTC recurrence.

## Introduction

Papillary thyroid cancer (PTC), the most common endocrine malignancy, accounts for 90% of thyroid cancer which has the seventh increase in new cases of cancer in women (1). Although it has low mortality, PTC, in some cases, can develop into progressive invasive primary disease. Ten percent to 30% of patients experience tumor recurrence and even distant metastasis, especially 10 years after initial treatment (2, 3). Most recurrences require additional surgical intervention with increased psychological stress to patients and expense (4). Patients with aggressive PTC who are resistant to standard treatments may benefit from immunotherapy (5).

Immune responses against thyroid carcinoma have long been recognized (6, 7), evidenced by the frequent existence of lymphocytes within primary thyroid tumor and tumor surrounding areas (8). CD4^+^T, CD8^+^T, B, NK, and regulatory T (Treg) cells have been reported to be present in nodular goiter (NG) (9–11) and play different roles during thyroid tumor progression (12, 13). CD4^+^T and B cells are reported to be positively correlated with reduced tumor sizes in PTC (14). Increased tissue infiltration of Treg cells was positively correlated with advanced thyroid cancer stage, whereas NK-cell infiltration was negatively correlated, indicating that NK and Treg cells might be important regulators of PTC progression (10, 11, 15). High tumor-infiltrating CD8^+^T cell density was associated with a favorable prognosis in thyroid cancer patients (14, 16). On the other hand, a retrospective cohort study found that patients whose thyroid tumor samples were enriched in CD8^+^T cells present a poor outcome (17). Thus, the prognostic value of CD8^+^T cells in thyroid cancer is controversial, and the association of different CD8^+^T-cell subsets with PTC recurrence remains unclear. Moreover, in view of the differences in experimental methods and samples, the proportions of lymphocytes from PTC and MNG patients varied in different studies (9, 11, 15, 16, 18, 19). It is of great importance to profile the immune cells in tissues and peripheral blood of MNG and PTC patients.

Here, we delineated the specific immune landscape of multinodular non-toxic goiter (MNG) tissue and PTC tumor tissue *via* flow cytometry and multiplex immunohistochemistry. Interestingly, we found significant differences in phenotypes of thyroid infiltrated CD8^+^T cells between MNG and PTC patients. Particularly, PD-1^-^CD39^+^CD103^+^CD8^+^T, PD-1^+^CD39^-^CD103^+^CD8^+^T, and PD-1^+^CD39^+^CD103^-^CD8^+^T cells were associated with the recurrence of PTC.

## Materials and methods

### Patients

This study was performed following the regulations of the ethics committee of the second affiliated hospital of South China University of Technology. Thirteen multinodular non-toxic goiter (MNG) samples paired with peripheral blood samples and 17 papillary thyroid cancer (PTC) samples paired with paratumor and peripheral blood samples were analyzed by flow cytometry (**Table 1**). Thirteen MNG, 23 non-recurrent PTC, and eight recurrent PTC samples were subjected to multiplex immunohistochemistry (**Table 1**). Paratumor tissue was defined as 2~3 cm away from lesions. Thyroid tissue was collected and placed in 1640 medium containing 10% fetal bovine serum. PTC and MNG patients were confirmed by surgery and pathology. Patients with the following conditions were excluded: treated with chemoradiation therapy preoperatively; with hyperthyroidism, hypothyroidism, diabetes, hypertension and hyperlipidemia, or serum anti-thyroglobulin antibody (TGAb) or thyroid peroxidase antibody (TPOAb) levels higher than the reference range (TGAb >115 IU/ml, TPOAb >35 IU/ml); or with a background of thyroiditis. PTC recurrence was defined as recurrent or persistent disease based on authoritative histologic, cytologic, radiographic, or biochemical criteria (20). Patients with local, regional, and distant recurrences were all included.

**Table 1:** Demographics and clinicopathologic characteristics of PTC patients.

Characteristics	PTC (%)			MNG (%)
	FCM	mIHC		
		Recurrence	Non-recurrence	
**No. patients**	17	8	23	13
**Gender**				
Male	3 (17.65)	4 (50)	4 (17.39)	2 (15.38)
Female	14 (82.35)	4 (50)	19 (82.61)	11 (84.62)
**Age (years)**				
Mean ± SD	39.47 ± 11.09	49.5 ± 18.81	41.26 ± 12.34	48.92 ± 10.38
<55	15 (88.24)	4 (50)	18 (78.26)	8 (61.54)
≥55	2 (11.76)	4 (50)	5(21.74)	5 (38.46)
**Tumor size (cm)**				
<2	14 (82.35)	7 (87.5)	19 (82.61)	
≥2	3 (17.65)	1 (12.5)	4 (17.39)	
**T stage**				
T1				
T1a	11 (64.71)	1 (12.5)	16 (69.56)	
T1b	4 (23.53)	7 (87.5)	5 (21.74)	
T2	0	0	2 (8.70)	
T3	0	0	0	
T4				
T4a	1 (5.88)	0	0	
T4b	1 (5.88)	0	0	
**N stage**				
N0	10 (58.82)	0	11 (47.83)	
N1				
N1a	5 (29.41)	3 (37.5)	11 (47.83)	
N1b	2 (11.76)	5 (62.5)	1 (4.35)	
**M stage**				
M0	16 (94.12)	8 (100)	23 (100)	
M1	1 (5.88)	0	0	

SD, standard deviation.

### Data availability and calculation of microenvironment cell abundance

THCA RNA-seq expression profiles from TCGA database (https://portal.gdc.cancer.gov/) were downloaded using the GDC Data Transfer Tool Client (https://gdc.cancer.gov/access-data/gdc-data-transfer-tool). Custom Perl scripts (Perl version 5.8.9) used for processing the FPKM expression data are available on request. Four sets of datasets GSE197852 (21), GSE3467 (22), GSE33630 (23), and GSE6004 (24) from the GEO database were used to analyze Treg-cell subpopulations. Marker genes for microenvironment cells were obtained from previous studies (25–30). The 470 genes representing 18 microenvironment cell types are listed in **Supplementary Table 3**. We used single-sample gene set enrichment analysis (ssGSEA, “GSVA” function in R) to calculate the abundance of microenvironment cells in each sample. The CD8^hi^ samples were selected based on the mean level of CD8^+^T-cell marker gene expression and were considered to be enriched in CD8^+^T cells. These patients were separated into eight clusters based on the expression levels of PDCD1, ENTPD1, and ITGAE genes. PD-1^hi^, CD103^hi^, and CD39^hi^ were defined as higher than the mean expression levels of PDCD1, ENTPD1, and ITGAE in the CD8^hi^ samples, respectively, whereas PD-1^lo^, CD103^lo^, and CD39^lo^ were the opposite.

### Mononuclear cell isolation

Tumor or paratumor tissues from PTC and control thyroid tissue from MNG were washed with precooled saline. Tissues were cut into small pieces in a tube containing RPMI-1640 medium and 10% FBS on ice. Cells were collected by washing the thyroid tissues and filtering cell suspension through a 100-mesh strainer. After centrifugation at 450g for 5 min, cells were collected and red blood cells were depleted by adding 1–2 ml red blood cell lysis buffer (Beyotime, China) and incubating at 4°C for 5 min. Lymphoprep (Axis Shield, Norway) was used to isolate peripheral blood mononuclear cells (PBMCs) according to the manufacturer’s instructions. Peripheral blood was centrifuged to collect plasma and cells. The cells were diluted by addition of an equal volume of 0.9% saline. Diluted blood was layered over 3 ml Lymphoprep and centrifuged at 800g for 20 min at room temperature. Cells at the interface were collected and counted on a hemocytometer in the presence of trypan blue.

### Flow cytometry

For surface marker staining, mononuclear cells (MNCs) from tissue and blood were incubated with mouse serum for 15 min at 4°C, followed by fluorescent antibodies at 4°C for 20 min. BV421-conjugated anti-CD39 (A1), BV510-conjugated anti-CD3 (OKT3), BV605-conjugated anti-CD123 (6H6), BV650-conjugated anti-CD45RA (HI100), BV711-conjugated anti-CD14 (M5E2), BV785-conjugated anti-CD19 (HIB19), FITC-conjugated anti-CD45RO (UCHL1), PerCP-Cy5.5-conjugated anti-CD16 (3G8), PE-conjugated anti-CD25 (BC96), PE/Dazzle594-conjugated anti-PD-1 (EH12.2H7), PE/Cy5-conjugated anti-CD56 (5.1H11), Alexa700-conjugated anti-CD8a (HIT8a), PE/Cy7-conjugated anti-HLA-DR (LN3), APC-conjugated antibodies against CD103 (Ber-ACT8), and APC/Cy7-conjugated anti-CD45 (HI30) were purchased from BioLegend (San Diego, CA, USA). BUV563-conjugated anti-CD4 (SP3) and BUV737-conjugated anti-CD127 (HIL-7R-M21) were purchased from BD Biosciences (Franklin Lakes, New Jersey, USA). Dead cells were stained with DAPI (Beyotime, China). Live cells were gated as CD45^+^DAPI^−^. Data were acquired using a FACS LSRFortessa flow cytometer (BD Biosciences) and analyzed with the FlowJo (Tree Star, Ashland, OR, USA). Gating strategies for the flow experiment are described in **Figure S1**.

### t-Distributed stochastic neighbor embedding analysis

Live MNCs were gated in FlowJo to perform t-distributed stochastic neighbor embedding (t-SNE) analysis. Data from 10,000 cells of PBMCs or tissue MNCs were randomly selected and merged into one matrix and normalized by a channel using the scale function in R (version 3.5.3). Then, we ran the t-SNE algorithm by the RunTSNE function in the Seurat package (version 3.0.1) to output the results. The parameters used for t-SNE analysis were CD3, CD56, CD19, CD4, CD8, CD45RA, CD45RO, and CD14.

### Multiplex immunohistochemistry

Formalin-fixed paraffin-embedded tissues were cut into 4-μm slices and stained using the PANO 7-plex IHC kit (TSA-RM) (Panovue) according to the manufacturer’s instructions. Slides were deparaffinized with xylene and rehydrated with an ethanol gradient. Heat-induced antigen retrieval was performed in sodium citrate buffer (0.01M, pH = 6.0) before each primary antibody incubation, and the slides were cooled to room temperature. After deactivating the endogenous peroxidase with 3% H_2_O_2_ in methanol, the slices were blocked with 10% goat serum for 30 min followed by primary antibody staining. The primary antibodies and dilutions with PANO amplification diluent were applied in the following order: CD4 (ab133616, 1:500, Abcam) with Opal 620, CD20 (ab78237, 1:2,000, Abcam) with Opal 520, CD8 (C8/144B, 1:200, CST) with Opal 690, CD56 (123C3, 1:200, CST) with Opal 570, and CD45 (60287-1-Ig, 1:4,000, Proteintech) with Akoya 580. The CD8^+^T-cell subsets were labeled by CD8 (C8/144B, 1:200, CST) with Opal 690, CD103 (ab224202, 1:200, Abcam) with Opal 620, PD-1 (EH33, 1:200, CST) with Opal520, and CD39 (ab223842, 1:3000, Abcam) with Opal 540. All slides were incubated with PANO polymer HRP Ms+Rb for 15 min at room temperature. Nuclei were stained for 10 min with DAPI (Beyotime, China). All slides were scanned using the Vectra Automated Quantitative Pathology Imaging System (Vectra Polaris featuring MOTiF™), and images were analyzed with the HALO (version 3.1.1076) Digital Pathology system (Indica Labs).

### Statistical analysis

All data were presented as mean ± standard error of mean (SEM) and analyzed using GraphPad Prism 8.3 (San Diego, CA) and R (version 3.6.2). The comparison between two groups was performed with two-tailed Student’s t test (paired or unpaired) if both data conformed normal distribution and equality of variance in standard deviation. Otherwise, the non-parametric test (Wilcoxon signed-rank test or Mann–Whitney U test) was used. Recurrence-free survival (RFS) was analyzed with Kaplan–Meier estimates and log-rank tests. Cox proportional hazard regression models were constructed for clinicopathologic characteristics, CD8^hi^T-cell subsets, and RFS. Results were expressed as hazard ratio (HR) with 95% confidence interval (CI). Univariate logistic regression was used to estimate associations between clinical parameters and CD8^+^T subsets in tumor from flow cytometry and multiplex immunohistochemistry (mIHC) data, respectively. The relationships of PD-1^lo^CD39^hi^CD103^hi^ CD8^hi^T, PD-1^hi^CD39^hi^CD103^lo^ CD8^hi^T, and PD-1^hi^CD39^lo^CD103^hi^ CD8^hi^T cells in tumor from TCGA data with clinical parameters and other immune cells were analyzed using multinomial logistic regression analyses. Pearson’s chi-square test or Fisher’s exact test were employed for the comparison of unordered categorical variables. *p < 0.05; **p < 0.01; ***p < 0.001.

## Results

### Landscape of the immune microenvironment in thyroid tissues and peripheral blood

**Figure 1:**
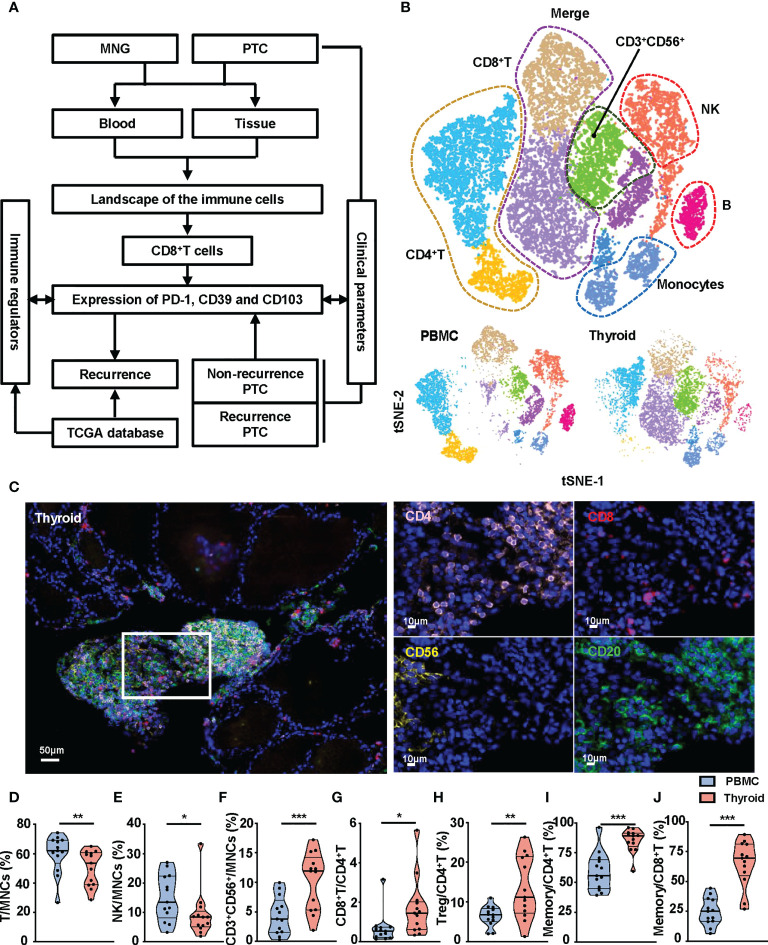
Composition of immune cell subsets in peripheral blood and thyroid tissues from MNG patients. **(A)** The flowchart of the work. **(B)** t-SNE islands for CD3^+^T cells, CD4^+^T cells, CD8^+^T cells, CD3^+^CD56^+^ cells, CD56^+^ NK cells, CD19^+^ B cells, and CD14^+^ monocytes of flow cytometry data. **(C)** Representative immunofluorescence staining of CD4 (pink), CD8 (red), CD56 (yellow), and CD20 (green) in an MNG. DAPI staining is shown in blue. Scale bar, 50 μm (left), 10 μm (right). Percentages of T cells **(D)**, NK cells **(E)**, and CD3^+^CD56^+^ cells **(F)** in MNCs in peripheral blood and thyroid tissue. **(G)** Ratio of CD8^+^T to CD4^+^T cells in peripheral blood and thyroid tissue. **(H)** Percentage of Treg cells in total CD4^+^T cells. Percentages of memory T cells (CD45RO^+^CD45RA^-^) in CD4^+^T **(I)** and CD8^+^T **(J)** cells. **(D–J)** The individual dot represents a patient, and data are presented as mean ± SEM. *p < 0.05; **p < 0.01; ***p < 0.001.

To explore the immune microenvironment of thyroid, we collected thyroid tissues and peripheral blood from MNG and PTC patients and performed flow cytometry and mIHC, in combination with bioinformatics analysis (**Figure 1A**). First, we used t-SNE maps to present the immune landscape of the thyroid and peripheral blood from MNG patients. Specific immune lineages were recognized using a color-based representation of expression levels of a single parameter. We identified six major cell clusters: CD8^+^T, CD4^+^ T, CD3^+^CD56^+^, NK, B cells, and monocytes (**Figures 1B**, **S2A**). We also used mIHC to detect the locations of these immune cell subsets in the thyroid. We found these immune cell subsets aggregated in the thyroid interfollicular space (**Figure 1C**). The frequencies and density of CD8^+^T, CD4^+^ T, NK, and B cells of MNG tissues obtained by mIHC were displayed (**Figures S2B, C**). The percentages of T and NK cells from thyroid tissues were decreased compared with peripheral blood (p = 0.0068 for T cells, p = 0.0134 for NK cells, **Figures 1D, E**). However, the percentage of CD3^+^CD56^+^ cells was significantly higher in thyroid tissues than in peripheral blood (p < 0.0001, **Figure 1F**). For T-cell subgroups in thyroid, the CD8^+^T/CD4^+^ T-cell ratio (p = 0.0171, **Figure 1G**) and the percentage of Treg cells (CD127^-^CD25^+^CD4^+^T cells, p = 0.0044, **Figure 1H**) were increased when compared with peripheral blood. Moreover, T cells presented increased memory CD4^+^T cells (CD45RO^+^CD45RA^-^CD4^+^T cells, p = 0.0005, **Figure 1I**) and memory CD8^+^T cells (CD45RO^+^CD45RA^-^CD8^+^T cells, p = 0.0002, **Figure 1J**) in thyroid tissues. The percentages of B cells (**Figure S2D**) and monocytes (**Figure S2E**), between two groups did not show a statistically significant difference. These results show diverse immune landscapes across MNG tissues and paired peripheral blood, especially T-cell subsets.

### Higher percentage of memory T cells and Treg cells in PTC

**Figure 2:**
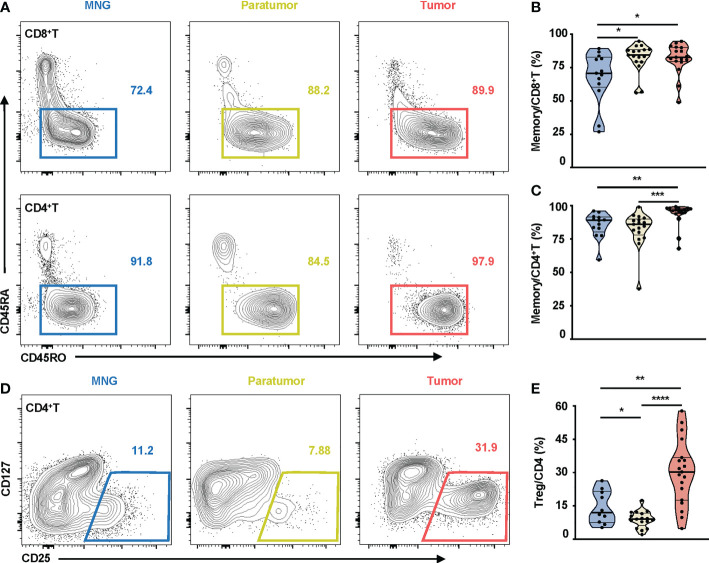
Memory T cells and Treg cells in thyroid tissues from MNG and PTC patients. **(A)** Representative FACS plots show CD45RO and CD45RA expressions on CD8^+^T and CD4^+^T cells. The values within solid line boxes indicate the proportions of memory T cells (CD45RO^+^CD45RA^-^) to CD8^+^T or CD4^+^T cells. **(B)** Frequencies of memory T cells relative to CD8^+^T cells are shown. **(C)** Frequencies of memory T cells relative to CD4^+^T cells are shown. **(D)** Representative FACS plots show CD25 and CD127 expressions on CD4^+^T cells. The values within solid line boxes indicate the proportions of Treg cells (CD25^+^CD127^-^) to CD4^+^T cells. **(E)** Frequencies of Treg cells relative to CD4^+^T cells are shown. Blue means MNG, yellow means paratumor, and red means tumor. **(B–E)** Data presented as mean ± SEM. *p < 0.05; **p < 0.01; ***p < 0.001; ****p < 0.0001.

There was no statistical difference in percentages of CD8^+^T, CD4^+^T, CD3^+^CD56^+^, NK, B cells, and monocytes between tumor, paratumor, and MNG tissues (**Figure S3A**), nor between the peripheral blood from PTC and MNG patients (**Figure S3B**). However, CD45RO was highly expressed on CD8^+^T and CD4^+^T cells in thyroid tissues (**Figure 2A**). Correspondingly, percentages of memory CD8^+^T cells were increased in tumor and paratumor as compared with MNG (p = 0.0216 for tumor *vs*. MNG, p = 0.0154 for paratumor *vs*. MNG), whereas a significant difference was not detected between tumor and paratumor (**Figure 2B**). Percentages of memory CD4^+^T cells were also significantly higher in tumor whether compared with MNG or paratumor, but there was no difference between paratumor and MNG (p = 0.0011 for tumor *vs*. MNG, p < 0.0001 for tumor *vs*. paratumor, **Figure 2C**). Furthermore, CD4^+^ T cells from PTC and MNG patients exhibited different expression levels of CD25 (**Figure 2D**). The percentage of Treg cells was increased in tumor compared with MNG or paratumor but decreased in paratumor when compared with MNG (p = 0.0012 for tumor *vs*. MNG, p < 0.0001, for tumor *vs*. paratumor, p = 0.0284, for paratumor *vs*. MNG, **Figure 2E**). Furthermore, we found that the ssGSEA score of CD45RA^+^FoxP3^lo^CD25^++^ cells in MNG was higher than that in paratumor (p = 0.0008) and tumor (p = 0.0102). The ssGSEA score of CD45RA^-^FoxP3^lo^CD25^++^ cells was higher in MNG compared with paratumor (p = 0.0019). The ssGSEA scores of CD45RA^+^FoxP3^lo^CD25^++^ cells (p = 0.0016), CD45RA^-^FoxP3^hi^CD25^+++^ cells (p < 0.0001), or CD45RA^-^FoxP3^lo^CD25^++^ cells (p < 0.0001) in tumor were all higher than paratumor (**Figure S4**).

### Different expression patterns of PD-1, CD39, and CD103 on CD8^+^T cells in PTC

**Figure 3:**
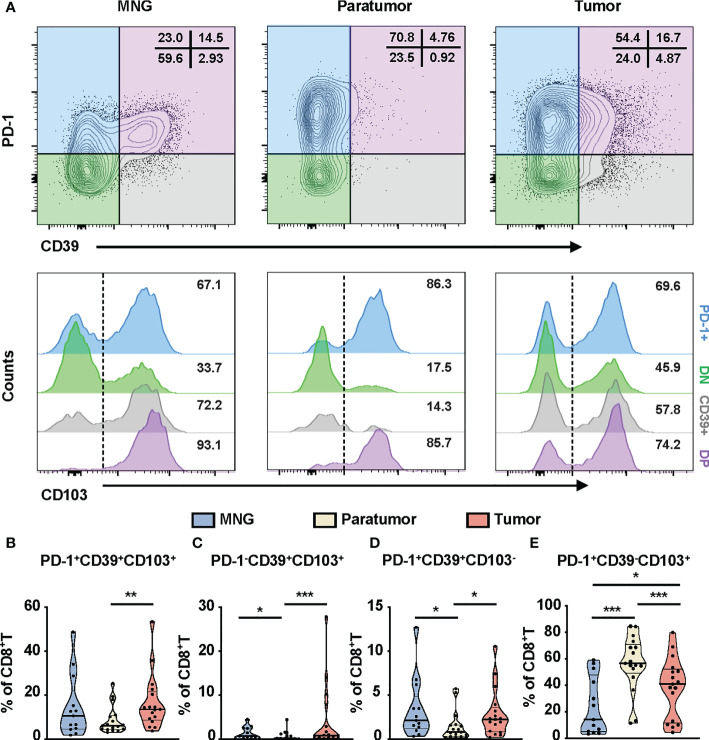
Phenotypic characteristics of CD8^+^T cells in thyroid tissues from MNG and PTC patients. **(A)** Flow cytometric analysis PD-1 and CD39 expression on CD8^+^T cells from thyroid tissues of MNG and PTC patients. Numbers in each quadrant indicate percent cells positive for PD-1 and/or CD39 on CD8^+^T cells. Below, histograms show the CD103 expression on PD-1^+^CD39^-^ (PD-1^+^, blue), PD-1^-^CD39^-^ (DN, green), PD-1^-^CD39^+^ (CD39^+^, gray), and PD-1^+^CD39^+^ (DP, purple) cells. Frequencies of PD-1^+^CD39^+^CD103^+^CD8^+^T **(B)**, PD-1^-^CD39^+^CD103^+^CD8^+^T **(C)**, PD-1^+^CD39^+^CD103^-^CD8^+^T **(D)**, and PD-1^+^CD39^-^CD103^+^CD8^+^T **(E)** cells in thyroid tissues of MNG and PTC patients. Data presented as mean ± SEM. *p < 0.05; **p < 0.01; ***p < 0.001.

The controversial prognostic value of CD8^+^T cells in thyroid cancer may be due to the complexity and heterogeneity of the CD8^+^T-cell landscape. PD-1, CD103, and CD39 have been independently proposed as markers of tumor-reactive CD8^+^T cells in various cancers, possessing a distinct prognostic implication (31–33). Thus, we investigated the clinical relevance of these CD8^+^T-cell subsets in PTC. First, based on the expressions of PD-1 and CD39, CD8^+^T cells from PTC and MNG tissues were divided into four subgroups (PD-1^+^: PD-1^+^CD39^-^, DN: PD-1^-^CD39^-^, CD39^+^: PD-1^-^CD39^+^, DP: PD-1^+^CD39^+^). These four subgroups were further divided into eight subsets according to the expression levels of CD103 (**Figure 3A**). Percentages of triple-positive subsets (p = 0.0067), PD-1^-^CD39^+^CD103^+^CD8^+^T (p = 0.0005), and PD-1^+^CD39^+^CD103^-^CD8^+^T cells (p = 0.0106) were higher in tumor than in paratumor (**Figures 3B–D**). The percentages of PD-1^-^CD39^+^CD103^+^CD8^+^T cells (p = 0.0133) and PD-1^+^CD39^+^CD103^-^CD8^+^T cells (p = 0.0174) from paratumor were lower than from MNG (**Figures 3C, D**). The percentage of PD-1^+^CD39^-^CD103^+^ cells in tumor was lower compared with paratumor (p < 0.0001) but higher than MNG (p = 0.0389, **Figure 3E**). In addition, the percentage of PD-1^+^CD39^-^CD103^+^CD8^+^T cells derived from paratumor was significantly increased compared with MNG (p = 0.0004, **Figure 3E**). Our univariate logistic regression analysis of CD8^+^T-cell subsets and clinical parameters indicated that big tumor size was a risk factor for high PD-1^-^CD39^+^CD103^+^CD8^+^T cells, but older age was a protective factor for high PD-1^+^CD39^-^CD103^+^CD8^+^T cells (**Table S1**). Together, these results revealed CD8^+^T-cell subsets may play different roles during PTC tumor development and progression.

### PD-1^+^CD39^+^CD103^+^CD8^+^T-cell and PD-1^+^CD39^+^CD103^-^CD8^+^T-cell frequencies are associated with recurrence of PTC patients

**Figure 4:**
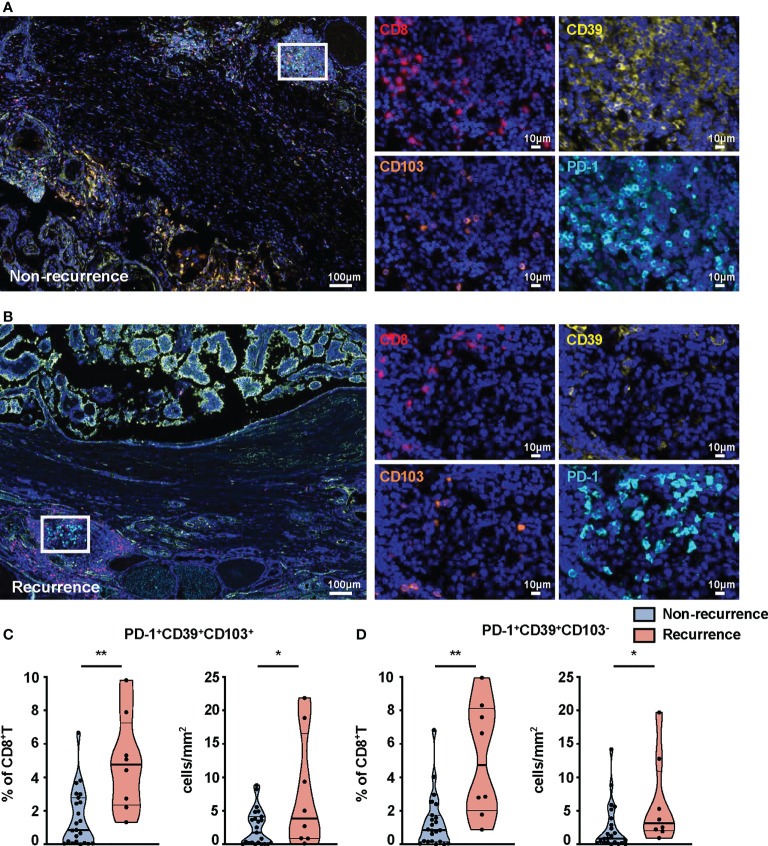
mIHC-based quantification of CD8^+^T-cell subsets in tissues from non-recurrent and recurrent PTC patients. Representative immunofluorescence staining of CD8 (red), CD39 (yellow), CD103 (orange), and PD-1 (cyan) in non-recurrent **(A)** and recurrent PTC tissues **(B)**. DAPI staining is shown in blue. Scale bar, 100 μm, 10 μm. Frequencies **(C)** and density **(D)** of PD-1^+^CD39^+^CD103^+^CD8^+^T cells and PD-1^+^CD39^+^CD103^-^CD8^+^T cells in non-recurrent PTC tissues (n = 23) and recurrent PTC tissues (n = 8). (Density: total PD-1^+^CD39^+^CD103^+^CD8^+^T- and PD-1^+^CD39^+^CD103^-^CD8^+^T-cell numbers divided by area of tissue per paraffin sections). Data presented as mean ± SEM. *p < 0.05; **p < 0.01.

We further compared the percentages of these subsets in tumors from recurrent and non-recurrent PTC patients by mIHC. Immunofluorescence staining of CD8^+^T-cell subset markers (PD-1, CD39, CD103, CD8) was used to calculate their number in the PTC (**Figures 4A, B**). The percentages (p = 0.0015) and density (p = 0.0251) of PD-1^+^CD39^+^CD8^+^T cells were both significantly higher in recurrence tumor than in non-recurrence tumor (**Figure S5A**). Furthermore, the percentages of PD-1^+^CD39^+^CD103^+^CD8^+^T (p = 0.0022) and PD-1^+^CD39^+^CD103^-^CD8^+^T (p = 0.001) cells were significantly higher in recurrence tumor than non-recurrence tumor, and this phenomenon was seen for the density of these two CD8^+^T-cell subsets (p = 0.0178 for PD-1^+^CD39^+^CD103^+^CD8^+^T cells, p = 0.0332, for PD-1^+^CD39^+^CD103^-^CD8^+^T cells, **Figures 4C, D**). As for other CD8^+^T-cell subsets, there was no significant difference in the two groups of PTC patients (**Figures S5B–G**). It was notable that no difference between tumors with recurrence and without recurrence was observed in the number and density of CD8^+^T cells (data not shown). Logistic regression analysis also demonstrated that PD-1^+^CD39^+^CD103^+^CD8^+^T (OR = 13.125, 95% CI: 1.876–268.997, p = 0.026) and PD-1^+^CD39^+^CD103^-^CD8^+^T (OR = 13.125, 95% CI: 1.876–268.997, p = 0.026) cells in tumor were both associated with high risk of tumor recurrence, despite no association found between these two CD8^+^T subsets and clinical parameters (**Table S2**). These results confirmed that the prominent infiltration of PD-1^+^CD39^+^CD103^+^CD8^+^T and PD-1^+^CD39^+^CD103^-^CD8^+^T cells was associated with relapse of PTC.

### CD8^+^T-cell subset signatures are associated with clinical features and recurrence of PTC patients

**Figure 5:**
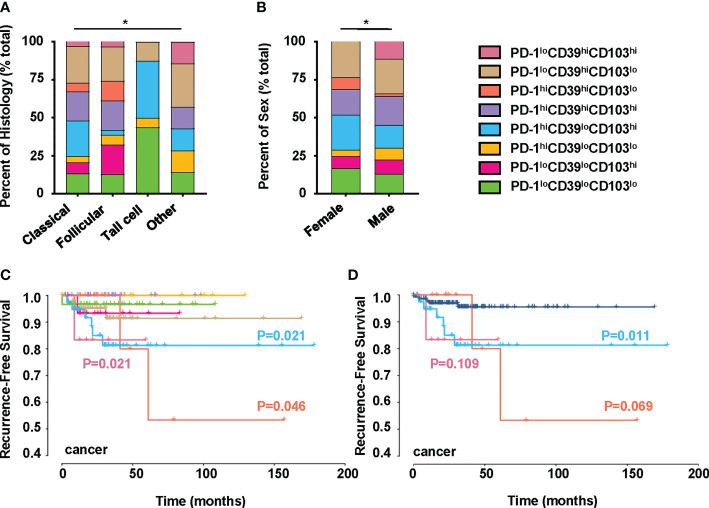
Clinical significance of PTC-infiltrating CD8^hi^T-cell subsets. This figure refers to TCGA THCA cohort (n = 195). **(A)** Comparison of pathological subtypes with CD8^hi^T-cell subsets. **(B)** Comparison of gender with CD8^hi^T-cell subsets. **(C)** The recurrence-free survival compared among eight clusters in TCGA database, including the PD-1^lo^CD39^lo^CD103^lo^ (n = 31), PD-1^lo^CD39^lo^CD103^hi^ (n = 16), PD-1^hi^CD39^lo^CD103^lo^ (n = 10), PD-1^hi^CD39^lo^CD103^hi^ (n = 41), PD-1^hi^CD39^hi^CD103^hi^ (n = 34), PD-1^hi^CD39^hi^CD103^lo^ (n = 12), PD-1^lo^CD39^hi^CD103^lo^ (n = 45), and PD-1^lo^CD39^hi^CD103^hi^ (n = 6) clusters. **(D)** The recurrence-free survival compared between the PD-1^hi^CD39^lo^CD103^hi^, PD-1^hi^CD39^hi^CD103^lo^, and PD-1^lo^CD39^hi^CD103^hi^ clusters and the other five clusters. Analyses were performed with Kaplan–Meier estimates and two-sided log-rank tests. p < 0.05 was considered significant. *p < 0.05.

We also investigated the clinical relevance and prognostic significance of these CD8^+^T-cell subsets by employing TCGA database. Although the PD-1^hi^CD39^hi^CD8^hi^T-cell subset was not associated with clinical features and recurrence of PTC (**Figure S5H**), significant differences in histology (p = 0.0406) and sex (p = 0.0019) were observed among the eight clusters of CD8^hi^ patients (**Figures 5A, B**). Male (OR = inf, 95% CI: 9.86E+32-1.52E+35, p < 0.001) was a significant risk factor for high PD-1^lo^CD39^hi^CD103^hi^ CD8^hi^T cells, but no significant association was detected between the other two CD8^hi^T subsets and clinical parameters (**Table 2**). More importantly, the PD-1^hi^CD39^lo^CD103^hi^ (p = 0.021), PD-1^hi^CD39^hi^CD103^lo^ (p = 0.046), and PD-1^lo^CD39^hi^CD103^hi^ (p = 0.021) clusters showed a higher recurrence risk in CD8^hi^ samples as compared with the PD-1^hi^CD39^hi^CD103^hi^ cluster (**Figure 5C**). When other five clusters as a whole were compared with these three clusters, only the PD-1^hi^CD39^lo^CD103^hi^ cluster still had a greater recurrence risk (p = 0.011, **Figure 5D**). The multivariate Cox proportional hazard model also revealed that the PD-1^hi^CD39^lo^CD103^hi^ cluster independently predicted worse recurrence-free survival (HR = 3.600, 95% CI: 1.070–12.110, p = 0.0385, **Table 3**).

**Table 2:** Multinomial logistic regression analysis of clinical parameters associated with PD-1^lo^CD39^hi^CD103^hi^ CD8^hi^T, PD-1^hi^CD39^hi^CD103^lo^ CD8^hi^T, and PD-1^hi^CD39^lo^CD103^hi^ CD8^hi^T cells in tumor from TCGA data.

PD-1^lo^CD39^hi^CD103^hi^ CD8^hi^T cell	OR (95% CI)	P
Age	0.866 (0.733-1.023)	0.091
Sex male	inf (9.86E+32-1.52E+35)	**0.000**
N stage		
N1	0.289 (0.008-10.324)	0.496
N1a	0	NA
N1b	0.803 (0.030-21.511)	0.896
T stage		
T1a	0	NA
T1b	2.09E+09(2.09E+09-2.09E+09)	**0.000**
T2	inf (4.97E+13-7.30E+15)	**0.000**
T3	inf (1.39E+13-8.03E+14)	**0.000**
T4	0	NA
Extrathyroidal extension	4.326 (0.088-212.915)	0.461
Recurrence	792.944 (1.438-437195.562)	**0.038**
PD-1^hi^CD39^hi^CD103^lo^ CD8^hi^T cell	OR (95% CI)	P
Age	1.005 (0.095-1.057)	0.836
Sex male	0.339 (0.030-3.837)	0.382
N stage		
N1	0.525 (0.044-6.308)	0.611
N1a	4.139 (0.891-19.220)	0.070
N1b	0	NA
T stage		
T1a	0	NA
T1b	0.947 (0.102-8.810)	0.962
T2	0.834 (0.106-6.571)	0.863
T3	1.006 (0.085-11.960)	0.996
T4	0	NA
Extrathyroidal extension	1.617 (0.159-16.453)	0.685
Recurrence	20.924 (2.056-212.926)	**0.010**
PD-1^hi^CD39^lo^CD103^hi^ CD8^hi^T cell	OR (95% CI)	P
Age	0.993 (0.966-1.022)	0.645
Sex male	0.816 (0.303-2.202)	0.689
N stage		
N1	0.404 (0.113-1.441)	0.162
N1a	1.010 (0.365-2.798)	0.985
N1b	0.154 (0.029-0.805)	**0.027**
T stage		
T1a	4.816 (0.675-34.373)	0.117
T1b	1.936 (0.408-9.181)	0.405
T2	1.322 (0.285-6.132)	0.721
T3	1.104 (0.180-6.768)	0.915
T4	1.678 (0.063-44.893)	0.758
Extrathyroidal extension	1.487(0.385-5.751)	0.565
Recurrence	5.144 (1.259-21.015)	**0.023**

Bold values refers to the risk factors associated with clinical features and other immune cells for the three tumor patient subsets of PD-1^lo^CD39^hi^CD103^hi^CD8^hi^T, PD-1^hi^CD39^hi^CD103^lo^CD8^hi^T, PD-1^hi^CD39^lo^CD103^hi^CD8^hi^T cells, respectively.

**Table 3:** Univariate and multivariate Cox proportional hazard models for recurrence-free survival.

Variables	Univariate		Multivariate	
	**HR (95% CI)**	P	**HR (95% CI)**	P
**Age (years)**				
<55			Not included	
≥55	1.1209 (0.3502-3.588)	0.848		
**Gender**				
Female			Not included	
Male	1.6003 (0.5352-4.786)	0.400
**Histology**				
Classical				
Follicular	0.4654 (0.0595-3.64)	0.446	0.6673 (0.082-5.461)	0.7061
Other	0.000 (0-Inf)	0.998	0.000 (0-Inf)	0.998
Tall cell	3.783 (1.0237-13.98)	**0.046**	4.549 (1.159-17.859)	**0.0299**
**Stage**				
I			not include	
II	0.000 (0-Inf)	0.998
III	1.783 (0.5830-5.456)	0.310
IV+IVA	1.069 (0.1333-8.579)	0.95
**CD8^hi^T cell subsets**				
other 5 clusters				
PD-1^hi^CD39^hi^CD103^lo^	4.3422 (0.8408-22.42)	0.0796	4.998 (0.937-26.666)	0.0596
PD-1^hi^CD39^lo^CD103^hi^	4.2236 (1.2880-13.85)	**0.0174**	3.600 (1.070-12.110)	**0.0385**
PD-1^lo^CD39^hi^CD103^hi^	5.7237 (0.6632-49.40)	0.1126	8.909 (0.999-79.468)	0.0501

Bold values refers to the establishment of univariate and multivariate Cox proportional hazards models for recurrence-free survival. The P value less than 0.05 has a statistical difference.

### The immune environment of PTC patients with different CD8^+^T-cell subset signatures

**Figure 6:**
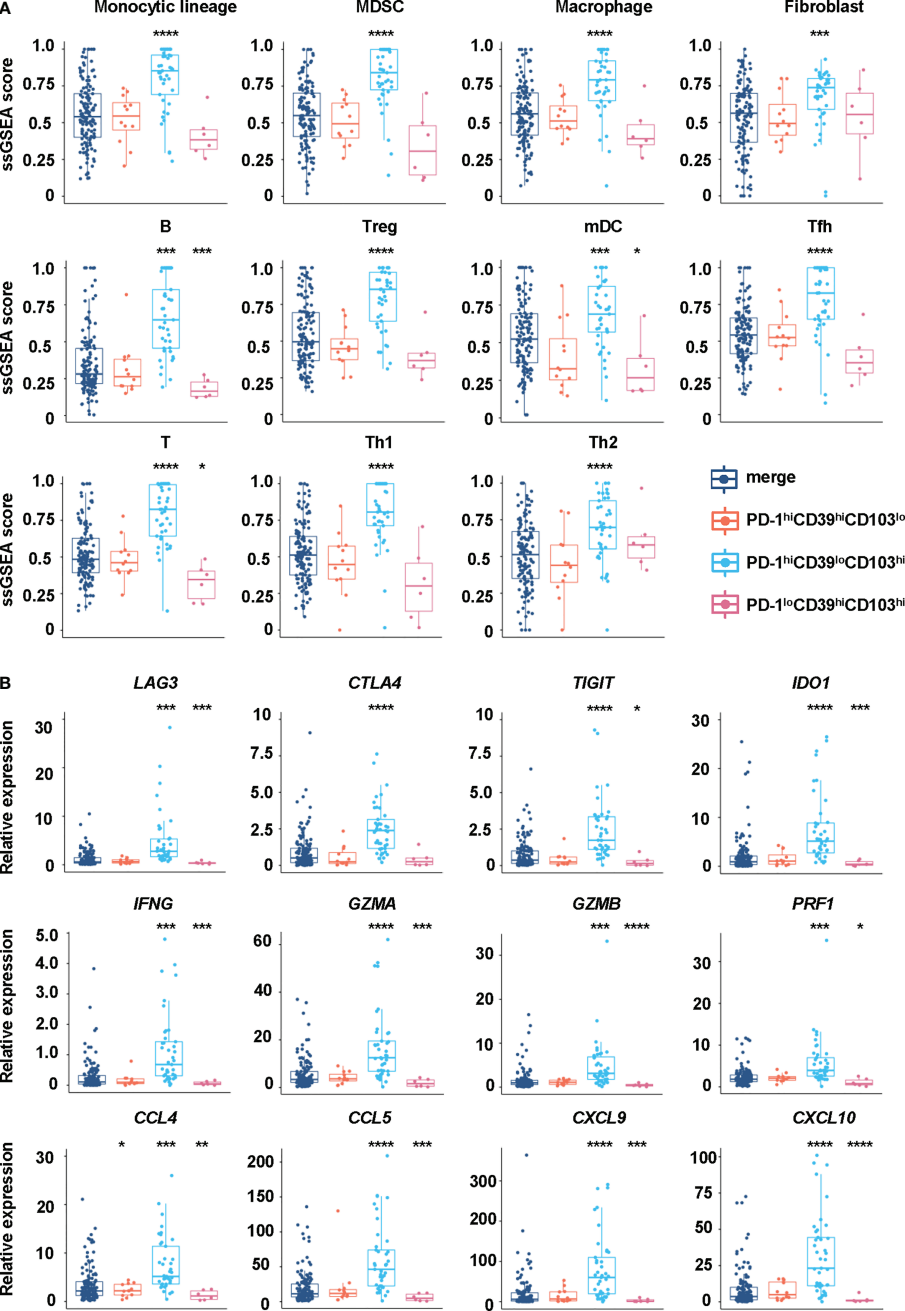
Differences in immune cells and regulatory factors in CD8^hi^T-cell subsets. **(A)** GSVA enrichment scores of the classical gene signatures for immune cells among the PD-1^hi^CD39^lo^CD103^hi^, PD-1^hi^CD39^hi^CD103^lo^, and PD-1^lo^CD39^hi^CD103^hi^ clusters and the other five clusters. **(B)** The mRNA expression of immune cell regulatory factors in the PD-1^hi^CD39^lo^CD103^hi^, PD-1^hi^CD39^hi^CD103^lo^, and PD-1^lo^CD39^hi^CD103^hi^ clusters compared with the other five clusters. *p < 0.05; **p < 0.01; ***p < 0.001; ****p < 0.0001.

We also analyzed the immune environment in the PD-1^hi^CD39^lo^CD103^hi^, PD-1^hi^CD39^hi^CD103^lo^, and PD-1^lo^CD39^hi^CD103^hi^ clusters and compared them with the other five clusters. We found that the PD-1^hi^CD39^lo^CD103^hi^ subset was markedly enriched in the monocytic lineage (p < 0.0001), myeloid-derived suppressor cells (MDSC, p < 0.0001), macrophage (p < 0.0001), fibroblasts (p < 0.0001), B cells (p < 0.001), regulatory T cells (Treg, p < 0.0001), myeloid dendritic cells (mDC, p < 0.0001), T follicular helper cells (Tfh, p < 0.0001), T cells (p < 0.0001), type 1 T helper cells (Th1, p < 0.0001), and type 2 T helper cells (Th2, p < 0.0001) (**Figure 6A**). Multinomial logistic regression analysis results also showed that the PD-1^hi^CD39^lo^CD103^hi^ subset was associated with the monocytic lineage (OR = 202.519, 95% CI: 27.920–1469.000, p < 0.001), MDSC (OR = 631.485, 95% CI: 65.345–6102.553, p < 0.001), macrophage (OR = 133.061, 95% CI: 18.424–960.976, p < 0.001), fibroblast (OR = 19.328, 95% CI: 3.085–121.090, p = 0.002), B cells (OR = 319.830, 95% CI: 46.454–2201.962, p < 0.001), Treg cells (OR = 123.978, 95% CI: 20.187–761.406, p < 0.001), mDC (OR = 11.857, 95% CI: 2.552–55.092, p = 0.002), Tfh cells (OR = 212.966, 95% CI: 28.659–1582.536, p < 0.001), T cells (OR = 545.290, 95% CI: 69.589–4272.833, p < 0.001), Th1 cells (OR = 413.319, 95% CI: 51.615–3309.738, p < 0.001), and Th2 cells (OR = 32.775, 95% CI: 5.808–184.943, p < 0.001) (**Table 4**). However, B cells (p < 0.001), mDC (p = 0.044), and T cells (p = 0.011) were decreased in the PD-1^lo^CD39^hi^CD103^hi^ cluster than in the other five clusters (**Figure 6A**). There was no statistical difference in the scores of neutrophil, NK cell, and type 17 T helper cell (Th17) among these four CD8^+^T-cell subsets (**Figure S6**). There was a decreased trend of mDC (p = 0.068) in the PD-1^hi^CD39^hi^CD103^lo^ cluster than in the other five clusters, whereas the difference did not reach statistical significance (**Figure 6A**). Of note, compared with the other five clusters, the PD-1^hi^CD39^lo^CD103^hi^ subset was highly enriched in immune checkpoint molecules (*LAG3*, *CTLA4*, *TIGIT*, *IDO1*, p < 0.001 for all molecules), cytotoxic molecules (*GZMA*, *GZMB*, *PRF1*, *IFNG*, p < 0.001 for all molecules), and chemokines (*CCL4*, *CCL5*, *CXCL9*, *CXCL10*, p < 0.001 for molecules). However, these genes in the PD-1^lo^CD39^hi^CD103^hi^ subset were in a low-expression state (**Figure 6B**). These results indicate that there are different mechanisms of CD8^+^T-cell subsets in PTC recurrence.

**Table 4:** Multinomial logistic regression analysis of immune cells associated with PD-1^hi^CD39^hi^CD103^lo^ CD8^hi^T, PD-1^hi^CD39^lo^CD103^hi^ CD8^hi^T, and PD-1^lo^CD39^hi^CD103^hi^ CD8^hi^T cells in tumor from TCGA data.

PD-1^hi^CD39^hi^CD103^lo^ CD8^hi^T cell	OR (95% CI)	P
Monocytic lineage	0.510 (0.033-8.014)	0.632
Myeloid dendritic cells	0.066 (0.004-1.120)	0.060
Neutrophil	0.557 (0.037-8.385)	0.673
B cells	0.260 (0.004-15.738)	0.520
Fibroblast	1.071 (0.089-12.892)	0.957
Macrophage	0.598 (0.035-10.305)	0.724
MDSC	0.399 (0.025-6.344)	0.515
NK cells	0.913 (0.055-15.033)	0.949
Regulatory T cells	0.156 (0.008-3.229)	0.230
T cells	0.356 (0.013-9.504)	0.538
T follicular helper cell	0.695 (0.036-13.567)	0.810
Type 1 T helper cell	0.204 (0.012-3.503)	0.273
Type 17 T helper cell	0.321 (0.020-5.261)	0.426
Type 2 T helper cell	0.283 (0.021-3.831)	0.343
PD-1^hi^CD39^lo^CD103^hi^ CD8^hi^T cell	OR (95% CI)	P
Monocytic lineage	202.519 (27.920-1469.000)	**0.000**
Myeloid dendritic cells	11.857 (2.552-55.092)	**0.002**
Neutrophil	1.834 (0.355-9.479)	0.469
B cells	319.830 (46.454-2201.962)	**0.000**
Fibroblast	19.328 (3.085-121.090)	**0.002**
Macrophage	133.061 (18.424-960.976)	**0.000**
MDSC	631.485 (65.345-6102.553)	**0.000**
NK cells	0.911 (0.173-4.789)	0.913
Regulatory T cells	123.978 (20.187-761.406)	**0.000**
T cells	545.290 (69.589-4272.833)	**0.000**
T follicular helper cell	212.966 (28.659-1582.536)	**0.000**
Type 1 T helper cell	413.319 (51.615-3309.738)	**0.000**
Type 17 T helper cell	2.792 (0.527-14.795)	0.228
Type 2 T helper cell	32.775 (5.808-184.943)	**0.000**
PD-1^lo^CD39^hi^CD103^hi^ CD8^hi^T cell	OR (95% CI)	P
Monocytic lineage	0.033 (0.000-2.575)	0.125
Myeloid dendritic cells	0.009 (0.000-0.839)	**0.042**
Neutrophil	0.085 (0.002-3.217)	0.184
B cells	0.000 (0.000-0.445)	**0.035**
Fibroblast	1.081 (0.034-34.036)	0.965
Macrophage	0.056 (0.001-3.689)	0.177
MDSC	0.008 (0.000-0.632)	**0.030**
NK cells	0.011 (0.000-0.386)	**0.013**
Regulatory T cells	0.025 (0.000-3.527)	0.144
T cells	0.000 (0.000-0.243	**0.017**
T follicular helper cell	0.011 (0.000-1.130)	0.056
Type 1 T helper cell	0.007 (0.000-0.568)	**0.027**
Type 17 T helper cell	0.009 (0.000-0.541)	**0.024**
Type 2 T helper cell	6.731 (0.171-264.189)	0.309

Bold values refers to the risk factors associated with clinical features and other immune cells for the three tumor patient subsets of PD-1^lo^CD39^hi^CD103^hi^CD8^hi^T, PD-1^hi^CD39^hi^CD103^lo^CD8^hi^T, PD-1^hi^CD39^lo^CD103^hi^CD8^hi^T cells, respectively.

## Discussion

In this study, multinodular non-toxic goiter (MNG), a benign disease with normal function of the thyroid gland, was taken as normal tissue to delineate the immune microenvironment, because completely normal thyroid tissue could not be obtained. We found that mononuclear cells distributed in clusters around the thyroid follicles and the percentages of mononuclear cells in MNG tissues were altered as compared with peripheral blood, especially the increase in memory T cells. During thyroid tumorigenesis, more memory T cells and Treg cells were detected in PTC tumor tissues. By delineating the immune microenvironment of MNG tissues, relatively normal thyroid tissues, and comparing them with PTC, we illustrated that CD8^+^T cells exhibited distinct activation patterns according to PD-1, CD39, and CD103 expression profiles in PTC, which were correlated with PTC relapse.

In line with the previous studies (11), we detected approximately 30.55% CD8^+^T (**Figure S2F**) and 62.32% CD4^+^T (**Figure S2G**) cells in PBMC of MNG. However, the percentages of several cell subsets in our observations differ markedly from those in a previous study (34), including 59.18% *vs*. 18.74% for memory CD4^+^T cells (**Figure 1I**), 23.16% *vs*. 42.8% for memory CD8^+^T cells (**Figure 1J**), and 9.61% *vs*. 12.7% for B cells (**Figure S2D**) in PBMC. We speculated that this may be due to differences in the method of peripheral blood immune cell preparation. Consistent with the results of Gogali et al. (11), we found the Treg cell and CD8^+^T/CD4^+^T cell ratio especially memory T cells in thyroid tissues significantly higher than peripheral blood of MNG patients, whereas that in NK cells was the opposite (**Figures 1E–J**), indicating the activation of antigen-specific naïve T cells in thyroid tissue and migration of memory T cells through blood and home to inflamed tissue (35). CD3^+^CD56^+^ cells are a heterogeneous lymphoid population that recognizes the lipid antigens presented by CD1d and has both immune-enhancing and immunosuppressive roles (36). The proportion of CD3^+^CD56^+^ cells was increased in thyroid tissues than in peripheral blood. NKT cells have not been characterized in thyroid tissues. In this study, we summarized the percentage of immune cells from MNG, which was different from peripheral blood and mapped the lymphocyte cells in spatial positions.

Consistent with previous studies (9, 11), we found no significant differences in the percentages of CD8^+^T, CD4^+^T, and NK cells in PTC tissues compared with MNG tissues (**Figure S3A**) and in PTC peripheral blood compared with MNG peripheral blood (**Figure S3B**). The difference in the percentages of CD8^+^T and CD4^+^T cells between tumor and paratumor tissues also had no statistical significance (**Figure S3A**). However, in TCGA database, the proportion of CD8^+^T cells was significantly decreased (16), whereas the proportion of CD4^+^T cells from tumor tissues was increased as compared with that from paratumor (37). It is well known that memory T cells are antigen-specific T cells that typically express CD45RO and can rapidly differentiate into effector T cells to kill the target cells once encountering the same antigen again (38). Therefore, increased memory T cells in PTC reflect that the immune response has been fully activated. Paratumor had more memory CD8^+^T cells compared with MNG, suggesting that the immune microenvironment of the thyroid lobe with tumor involvement may have already altered. Our data displayed that there were differences in Treg cells between MNG, paratumor, and tumor, with the highest proportion of tumor, which was consistent with other PTC studies (11, 19). Notably, the proportion of Treg cells in paratumor was the lowest and distinct from MNG in our results. Human CD4^+^CD25^+^ Treg cells were composed of phenotypically and functionally distinct subpopulations, which could be separated by the combination of FoxP3 and CD45RA staining, including CD45RA^+^FoxP3^lo^CD25^++^ resting Treg cells (rTreg cells) and CD45RA^-^FoxP3^hi^CD25^+++^ activated Treg cells (aTreg cells), both of which were suppressive *in vitro*, and cytokine-secreting CD45RA^-^FoxP3^lo^CD25^++^ (non-suppressive Treg cells) (26). Our results showed that rTreg cells and non-suppressive Treg cells were dominant Treg-cell subsets in MNG (**Figure S4**). In addition, Treg cells can migrate into the tumor microenvironment *via* various chemokine–chemokine receptor pathways (39–43). Based on this, we speculated that Treg cells in paratumor migrated into tumor nests, resulting in lower Treg cells than MNG (**Figure 2E**). Altogether, once PTC had developed, T cells presented a more activated state, and the variations of paratumor and MNG were different.

Our results found that PD-1, CD39, and CD103 were expressed on the CD8^+^T-cell surface in MNG and PTC tissues and presented diverse phenotypes. PD-1 is primarily a marker of T-cell activation (44, 45), but the PD-1 pathway can also regulate T-cell responses during cancer, where persistent antigen stimulation can lead to T-cell exhaustion (46). PD-1 has also been reported to be highly expressed on the T-cell surface in PTC, which was related to the aggressiveness of the disease (47, 48). CD39 is a ectonucleotidase encoded by the ENTPD1 gene, which can catalyze the hydrolysis of eATP and ADP released due to inflammatory stimulation or cell damage into AMP, which is then used by CD73 to synthesize adenosine with immunosuppressive effects (49). CD103 is expressed on a population of T cells found among peripheral tissues, known as tissue-resident memory T cells (T_RM_) (50). CD8^+^T cells are important for PTC recurrence (17), but we found that only some PTC patients with a specific phenotype of CD8^+^T cells showed a high recurrence risk. In our study, not only did PD-1^+^CD39^+^CD103^-^CD8^+^T, PD-1^+^CD39^-^CD103^+^CD8^+^T, and PD-1^-^CD39^+^CD103^+^CD8^+^T cells increase in PTC (**Figures 3**, **4**) but also the increase in these three CD8^+^T-cell subsets, especially PD-1^+^CD39^-^CD103^+^CD8^+^T cells, predicted a poor recurrence-free survival of PTC patients (**Figure 5**). In lung cancer with a more advanced stage, the fractions of PD-1^+^CD39^+^CD8^+^T cells tend to be higher (51). PTC patients with PD-1^-^CD39^+^CD103^+^CD8^+^T cells had decreased B cells and mDC (**Figure 6A**), which are essential for CD8^+^T-cell activation and antitumor response (52, 53). In addition, our research showed that CD8^+^T cells in these patients were also in a state of impaired function with low expressions of cytotoxic molecules and perforin, which is critical for antitumor immunity (46) (**Figure 6B**). Interestingly, PTC patients with PD-1^+^CD39^-^CD103^+^ CD8^+^T cells were enriched in immune cells and had a high expression of inhibitory immune checkpoints and chemokines (**Figure 6**, **Table 4**), which is similar with the phenotype of anaplastic thyroid carcinoma-like PTC (54). The anaplastic thyroid carcinoma-like tumors are hot and altered–immunosuppressed tumors, indicating that PD-1^+^CD39^-^CD103^+^CD8^+^T cells may be in a dysfunctional state and exert immune suppressive potential in PTC progression. Although BRAF^V600E^ mutation is related to CD8^+^T cells in PTC (16), our data showed that CD8^+^T-cell subsets were not correlated with BRAF^V600E^ mutation (data not shown), only with histology and sex (**Figures 5A, B**). Herein, these specific CD8^+^T cells in PTC were detected to have an association with age, tumor size, or sex (**Tables 2**, **S1**), which are reported as risk factors for PTC recurrence (55–57).

In summary, we described the differences in immune cell composition between thyroid tissues and peripheral blood of MNG patients. More importantly, we compared the changes of immune cells in the process of MNG to PTC and discovered the changes in the active status of T cells after the occurrence of malignancy. We also revealed the heterogeneity for PTC based on the expressions of PD1, CD39, and CD103 on CD8^+^T cells. Although the detailed mechanisms need to be further elucidated in future studies, our findings have shown an important role of CD8^+^T-cell subsets in PTC rapid progression or recurrence, which might provide new ideas for the treatment of patients who dedifferentiate from differentiated thyroid cancer to anaplastic thyroid carcinoma.

## Data availability statement

The raw data supporting the conclusions of this article will be made available by the authors, without undue reservation.

## Ethics statement

The research was approved by Medical Ethics Committee of the second affiliated hospital of South China University of Technology (K-2019-185). The patients/participants provided their written informed consent to participate in this study. Written informed consent was obtained from the individual(s) for the publication of any potentially identifiable images or data included in this article.

## Author contributions

ZC, M-LG, and Y-YL performed the experiments. ZC and M-LG analyzed the data. KY and LL helped to analyze the mIHC results. FS and HG collected samples for the experiments. Q-ZL, BX, and Z-XL designed and provided the funding of the project. ZC wrote the manuscript. Q-ZL, LL, HG, BX, and Z-XL revised the manuscript. All authors contributed to the article and approved the submitted version.

## Funding

This work was supported by the Guangzhou Science and Technology Plan Project (202102080170).

## Acknowledgments

We would like to thank The Cancer Genome Atlas (TCGA) database for providing great help to our research.

## Conflict of interest

The authors declare that the research was conducted in the absence of any commercial or financial relationships that could be construed as a potential conflict of interest.

## Publisher’s note

All claims expressed in this article are solely those of the authors and do not necessarily represent those of their affiliated organizations, or those of the publisher, the editors and the reviewers. Any product that may be evaluated in this article, or claim that may be made by its manufacturer, is not guaranteed or endorsed by the publisher.

## Supplementary material

Supplementary Figure 1 Gating strategies for flow cytometry of peripheral blood **(A)** and tissue **(B)**. Mononuclear cells (MNCs, DAPI^-^CD45^+^CD16^-^) were gated from live leukocytes without doublets. T cells (CD3^+^CD56^-^), NK cells (CD3^-^CD56^+^) and CD3^+^CD56^+^ cells were identified from MNCs. B cells (CD19^+^) and monocytes (CD14^+^) were identified from CD3^-^CD56^-^ population. Memory T cells (CD45RO^+^CD45RA^-^) were gated separately on CD8^+^T and CD4^+^T cells. Treg cells were defined as CD127^-^CD25^+^CD4^+^T cells. Tissue CD8^+^T cells were further divided into eight subsets by PD-1, CD39 and CD103.

Supplementary Figure 2 Feature plots of t-SNE maps and comparison of CD8^+^T, CD4^+^T, B cells and monocytes in peripheral blood and thyroid tissues from MNG patients. **(A)** Feature plots of CD3, CD4, CD8, CD56, CD19, and CD14 expression. Frequencies **(B)** and density **(C)** of CD4^+^T (CD4^+^CD45^+^), CD8^+^T (CD8^+^CD45^+^), NK (CD56^+^CD45^+^) and B (CD20^+^CD45^+^) cells in CD45^+^ cells in MNG tissues by mIHC. Percentages of B cells **(D)** and monocytes **(E)** in MNCs in peripheral blood and thyroid tissue by flow cytometry. Percentages of CD8^+^T **(F)** and CD4^+^T **(G)** cells in T cells (CD3^+^ CD56^-^) in peripheral blood and thyroid tissue by flow cytometry. Data presented as mean ± SEM. * p < 0.05; ** p < 0.01; *** p < 0.001.

Supplementary Figure 3 Composition of immune cell subsets in MNG and PTC patients. **(A)** Percentages of CD8^+^T, CD4^+^T, CD3^+^CD56^+^, NK, B cells and monocytes in MNCs in MNG, paratumor, tumor tissue. **(B)** Percentages of CD8^+^T, CD4^+^T, CD3^+^CD56^+^, NK, B cells and monocytes in PBMC from MNG and PTC patients.

Supplementary Figure 4 Treg cell subsets in MNG and PTC patients. The ssGSEA score of CD45RA^+^FoxP3^lo^CD25^++^ cells **(A)**, CD45RA^-^FoxP3^hi^CD25^+++^ cells **(B)**, and CD45RA^-^FoxP3^lo^CD25^++^ **(C)** in MNG, paratumor and tumor tissues.

Supplementary Figure 5 mIHC-based quantification of immune cell subsets in tissues from MNG and PTC patients and clinical significance of PTC-infiltrating CD8^hi^T cell subsets based on the expression of PD-1 and CD39. Frequencies and density of PD-1^+^CD39^+^CD8^+^T **(A)**, PD-1^+^CD39^-^CD103^-^CD8^+^T **(B)**, PD-1^+^CD39^-^CD103^+^CD8^+^T **(C)**, PD-1^-^CD39^+^CD103^-^CD8^+^T **(D)**, PD-1^-^CD39^+^CD103^+^CD8^+^T **(E)**, PD-1^-^CD39^-^CD103^-^CD8^+^T **(F)** and PD-1^-^CD39^-^CD103^+^CD8^+^T **(G)** cells in non-recurrent PTC tissues (n=23) and recurrent PTC tissues (n=8) by mIHC. **(H)** Comparison of pathological subtypes, gender and the recurrence free survival among 4 clusters in TCGA database, including PD-1^hi^CD39^hi^ (n=46), PD-1^lo^CD39^lo^ (n=47), PD-1^lo^CD39^hi^ (n=51), PD-1^hi^CD39^lo^ (n=51) clusters.

Supplementary Figure 6 Enrichment scores of neutrophils, NK cells and Th17 cells in CD8^hi^T cell subsets. GSVA enrichment scores of the classical gene signatures for neutrophils **(A)**, NK cells **(B)** and Th17 cells **(C)** among PD-1^hi^CD39^lo^CD103^hi^, PD-1^hi^CD39^hi^CD103^lo^, PD-1^lo^CD39^hi^CD103^hi^ clusters and other 5 clusters.
