# Supplementary figures and images for: Immune profiling identifies CD8+ T-cell subset signatures as prognostic markers for recurrence in papillary thyroid cancer
Source: Front Immunol. 2022 Nov 7;13:894919. doi: 10.3389/fimmu.2022.894919 (PMC9676940; doi:10.3389/fimmu.2022.894919)

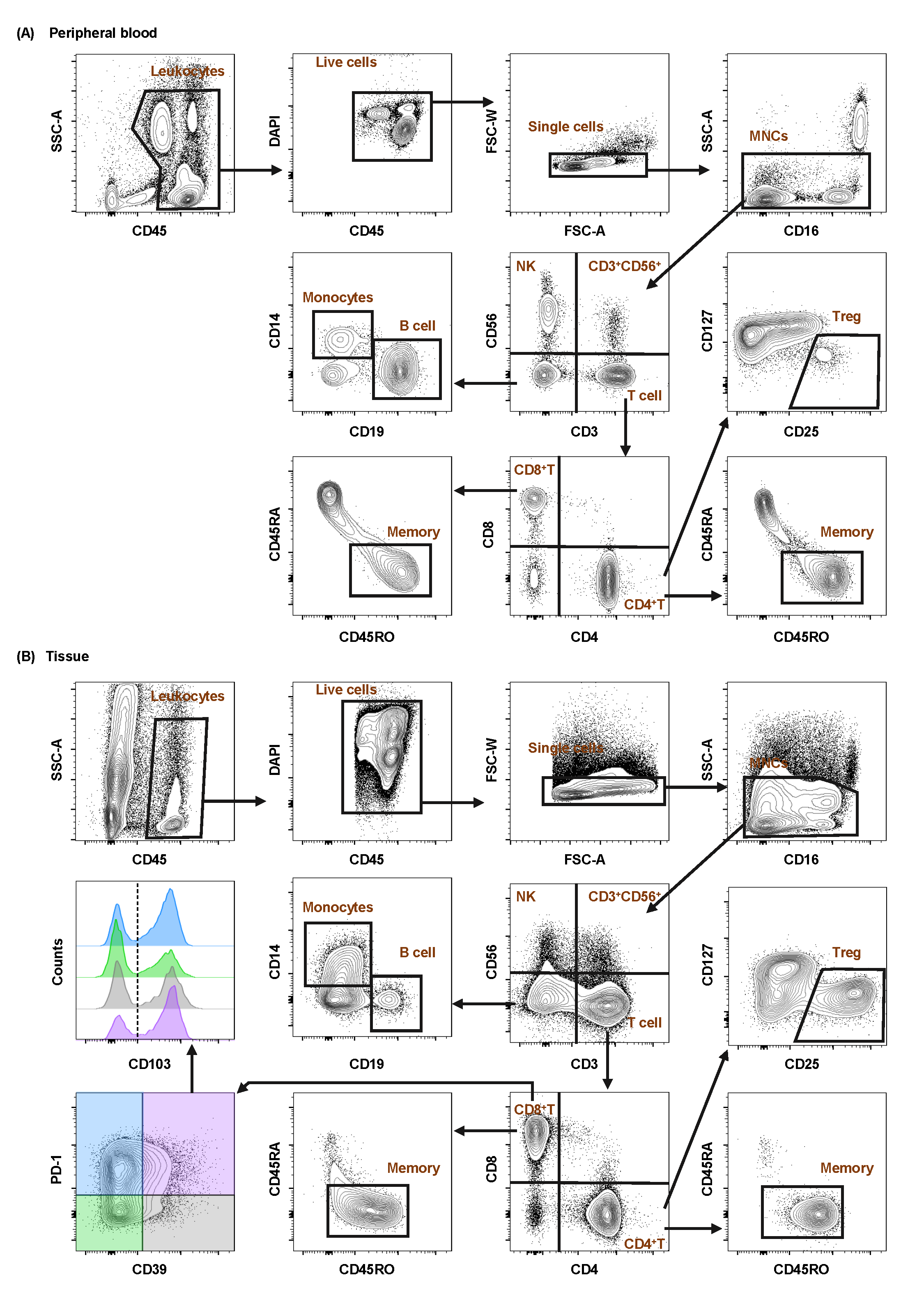

Supplement: Supplementary Figure 1 — Gating strategies for flow cytometry of peripheral blood (A) and tissue (B). Mononuclear cells (MNCs, DAPI-CD45+CD16-) were gated from live leukocytes without doublets. T cells (CD3+CD56-), NK cells (CD3-CD56+) and CD3+CD56+ cells were identified from MNCs. B cells (CD19+) and monocytes (CD14+) were identified from CD3-CD56- population. Memory T cells (CD45RO+CD45RA-) were gated separately on CD8+T and CD4+T cells. Treg cells were defined as CD127-CD25+CD4+T cells. Tissue CD8+T cells were further divided into eight subsets by PD-1, CD39 and CD103. [file Image_1.tiff]

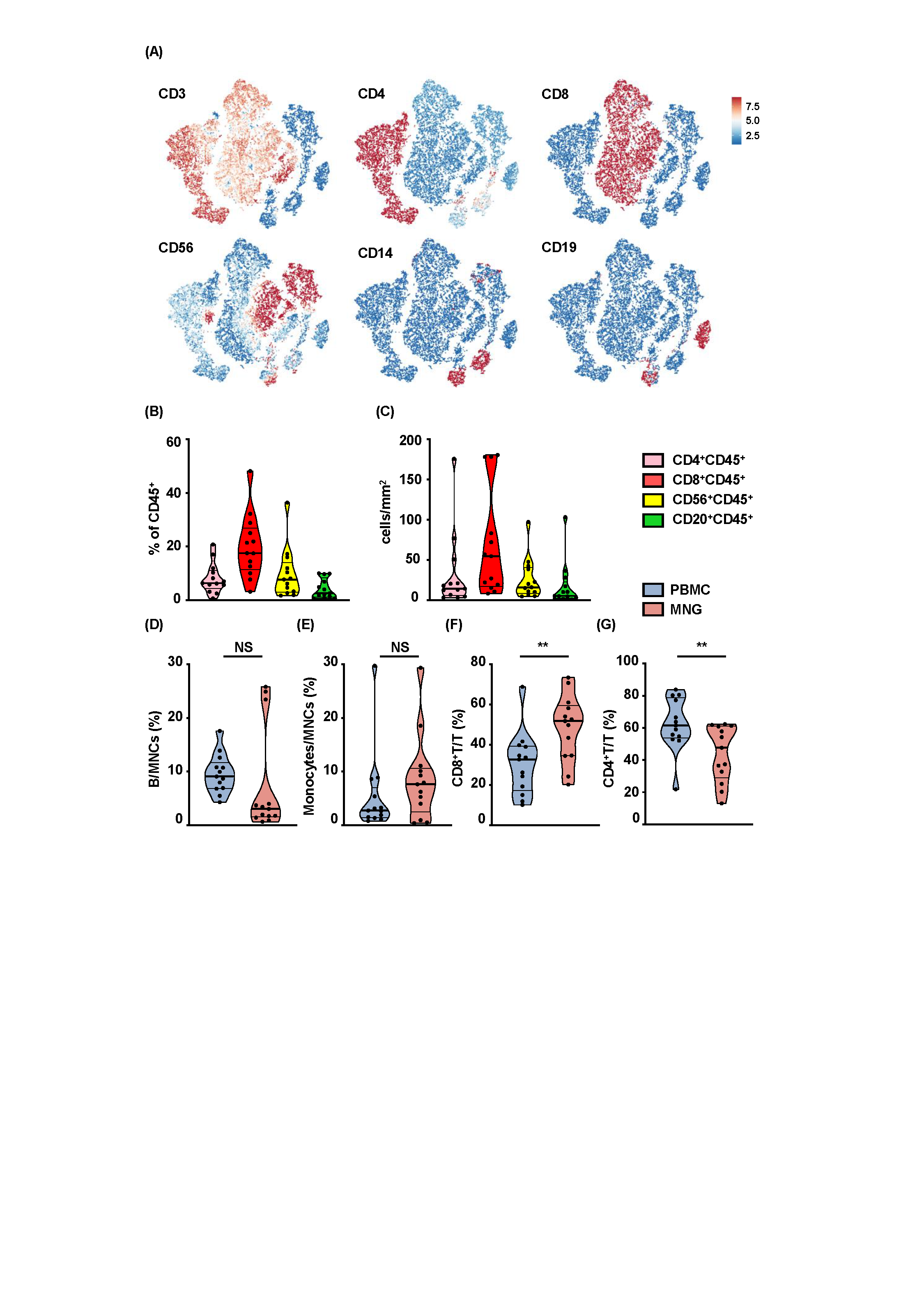

Supplement: Supplementary Figure 2 — Feature plots of t-SNE maps and comparison of CD8+T, CD4+T, B cells and monocytes in peripheral blood and thyroid tissues from MNG patients. (A) Feature plots of CD3, CD4, CD8, CD56, CD19, and CD14 expression. Frequencies (B) and density (C) of CD4+T (CD4+CD45+), CD8+T (CD8+CD45+), NK (CD56+CD45+) and B (CD20+CD45+) cells in CD45+ cells in MNG tissues by mIHC. Percentages of B cells (D) and monocytes (E) in MNCs in peripheral blood and thyroid tissue by flow cytometry. Percentages of CD8+T (F) and CD4+T (G) cells in T cells (CD3+ CD56-) in peripheral blood and thyroid tissue by flow cytometry. Data presented as mean ± SEM. * p < 0.05; ** p < 0.01; *** p < 0.001. [file Image_2.tiff]

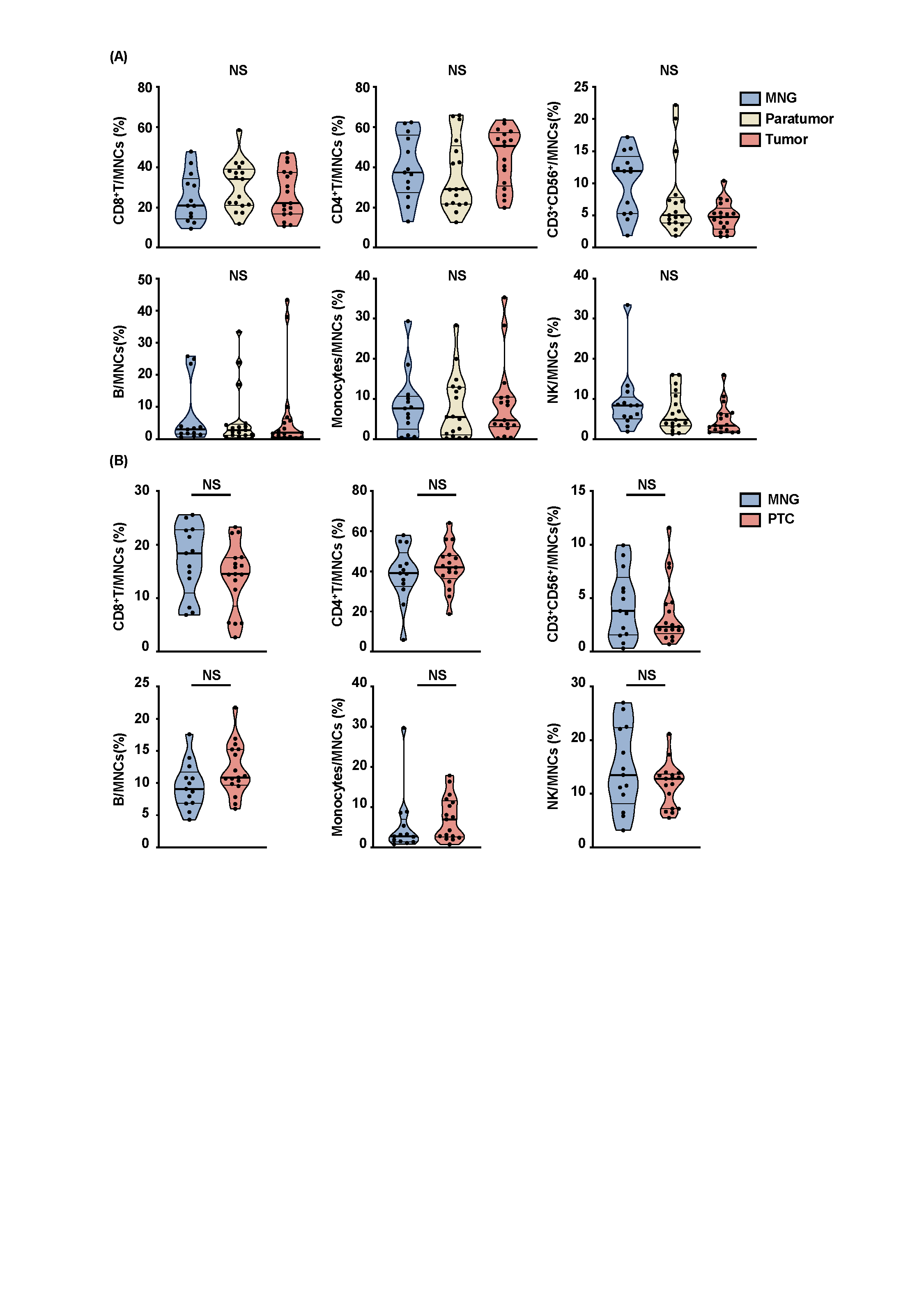

Supplement: Supplementary Figure 3 — Composition of immune cell subsets in MNG and PTC patients. (A) Percentages of CD8+T, CD4+T, CD3+CD56+, NK, B cells and monocytes in MNCs in MNG, paratumor, tumor tissue. (B) Percentages of CD8+T, CD4+T, CD3+CD56+, NK, B cells and monocytes in PBMC from MNG and PTC patients. [file Image_3.tiff]

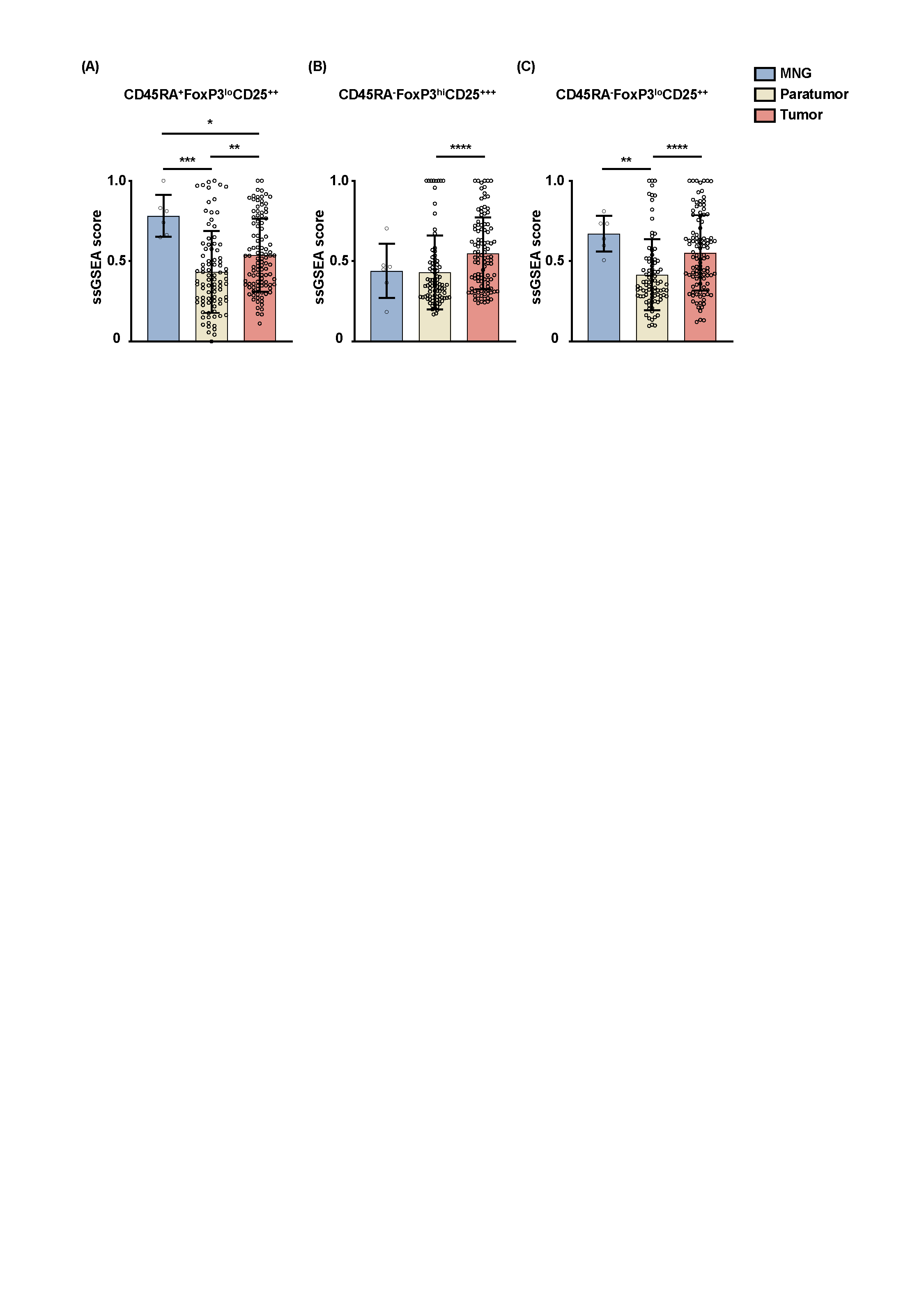

Supplement: Supplementary Figure 4 — Treg cell subsets in MNG and PTC patients. The ssGSEA score of CD45RA+FoxP3loCD25++ cells (A), CD45RA-FoxP3hiCD25+++ cells (B), and CD45RA-FoxP3loCD25++ (C) in MNG, paratumor and tumor tissues. [file Image_4.tiff]

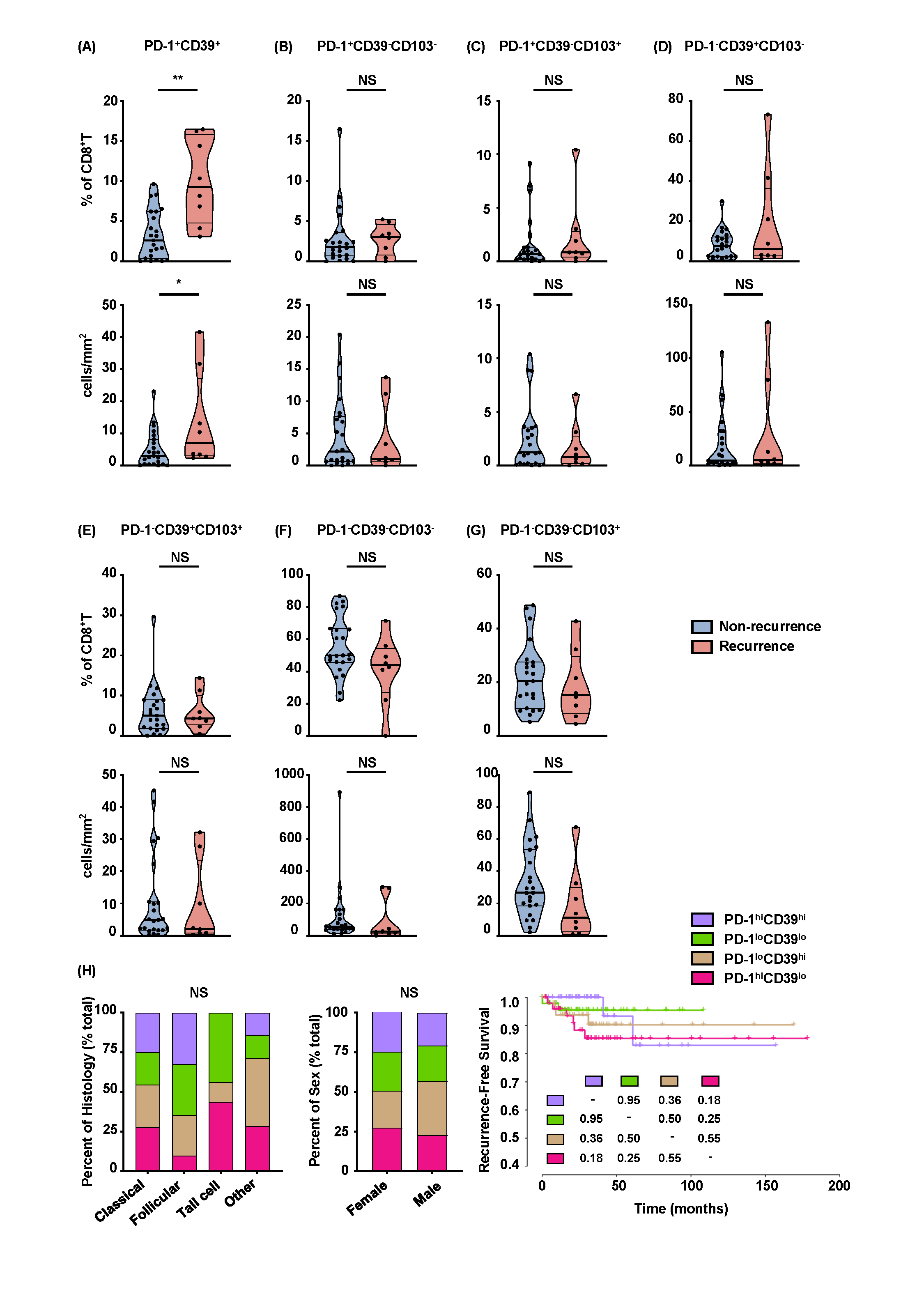

Supplement: Supplementary Figure 5 — mIHC-based quantification of immune cell subsets in tissues from MNG and PTC patients and clinical significance of PTC-infiltrating CD8hiT cell subsets based on the expression of PD-1 and CD39. Frequencies and density of PD-1+CD39+CD8+T (A), PD-1+CD39-CD103-CD8+T (B), PD-1+CD39-CD103+CD8+T (C), PD-1-CD39+CD103-CD8+T (D), PD-1-CD39+CD103+CD8+T (E), PD-1-CD39-CD103-CD8+T (F) and PD-1-CD39-CD103+CD8+T (G) cells in non-recurrent PTC tissues (n=23) and recurrent PTC tissues (n=8) by mIHC. (H) Comparison of pathological subtypes, gender and the recurrence free survival among 4 clusters in TCGA database, including PD-1hiCD39hi (n=46), PD-1loCD39lo (n=47), PD-1loCD39hi (n=51), PD-1hiCD39lo (n=51) clusters. [file Image_5.tiff]

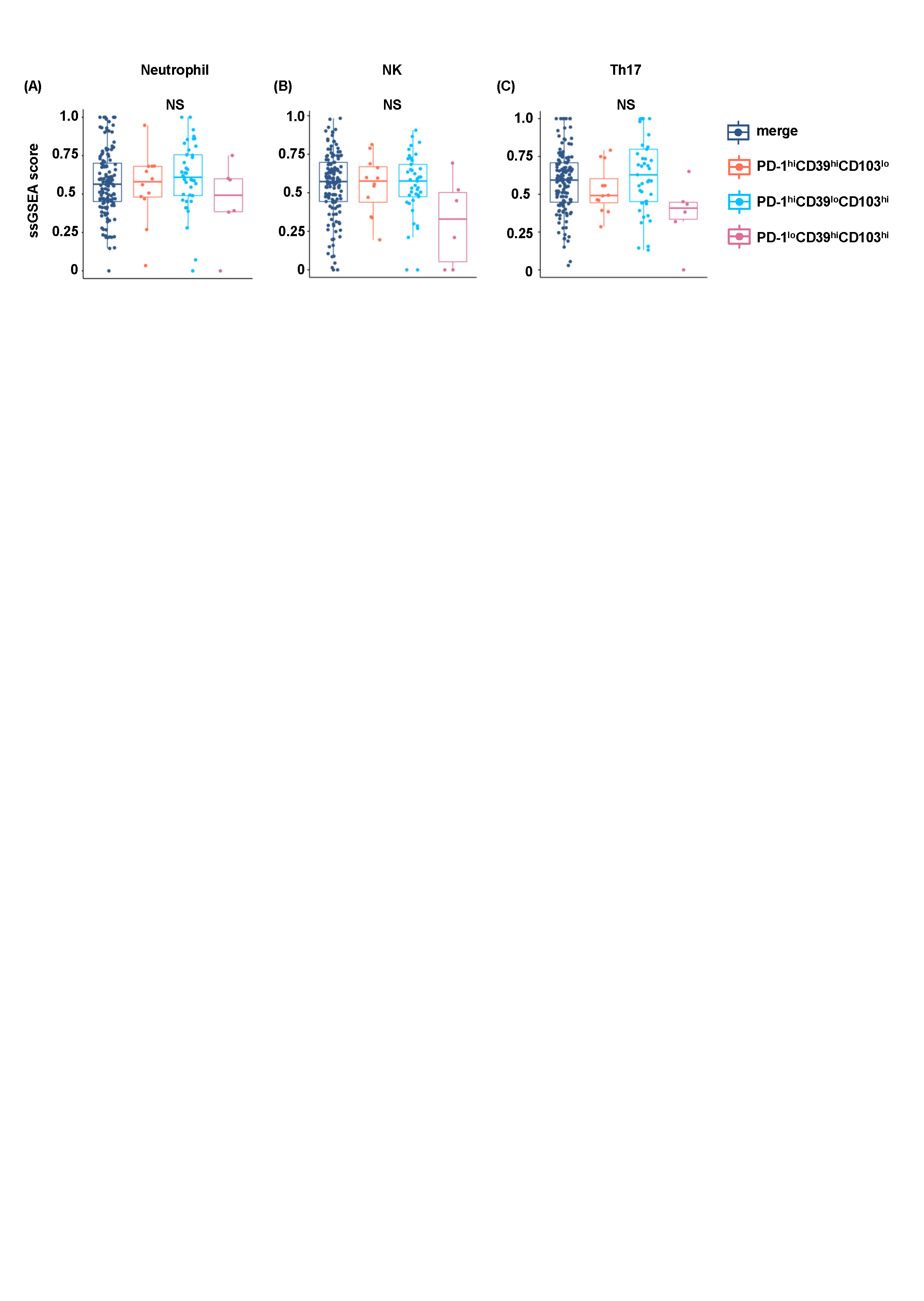

Supplement: Supplementary Figure 6 — Enrichment scores of neutrophils, NK cells and Th17 cells in CD8hiT cell subsets. GSVA enrichment scores of the classical gene signatures for neutrophils (A), NK cells (B) and Th17 cells (C) among PD-1hiCD39loCD103hi, PD-1hiCD39hiCD103lo, PD-1loCD39hiCD103hi clusters and other 5 clusters. [file Image_6.tiff]
